# Case Report: Treatment of Alectinib in NSCLC With Brain Metastasis Patient Refractory to Radiotherapy After Resistance to Crizotinib

**DOI:** 10.3389/fonc.2021.709188

**Published:** 2021-06-28

**Authors:** Chunzhi Zhang

**Affiliations:** Department of Radiation Oncology, Tianjin Hospital, Tianjin, China

**Keywords:** anaplastic lymphoma kinase, non-small-cell lung cancer, brain metastasis, radiotherapy, alectinib

## Abstract

**Background:**

Brain metastasis is the most common form of tumor recurrence after resistance to crizotinib in patients with anaplastic lymphoma kinase (ALK)-positive non-small-cell lung cancer (NSCLC). The treatment of brain metastasis in patients with ALK-positive NSCLC requires a multidisciplinary approach, including targeted therapy, chemotherapy, and radiotherapy. At present, no optimal treatment for these patients has been identified, although radiotherapy has remained a vital treatment.

**Case Presentation:**

We experienced a patient with ALK-positive NSCLC who developed brain metastasis after crizotinib therapy. ALK rearrangement was not detected in a blood sample using next-generation sequencing. In accordance with National Comprehensive Cancer Network guidance, the patient underwent whole-brain radiotherapy. However, the number of metastatic sites unexpectedly increased. In desperation, the patient was empirically given alectinib after radiotherapy failure, and unanticipated success was achieved.

**Conclusions:**

This case revealed some new insights. First, liquid biopsy is complementary to tissue biopsy in patients with NSCLC, mainly in those with EGFR mutation. However, ALK rearrangement should be assessed using tissue biopsy as much as possible. Second, brain metastasis of NSCLC might respond to second-generation tyrosine kinase inhibitors (TKIs), such as alectinib and ceritinib, after resistance to crizotinib regardless of the presence or absence of ALK rearrangement in liquid biopsy. Finally, combined radiotherapy and TKI therapy appears optimal in patients with brain metastasis of NSCLC after resistance to crizotinib in the absence of a definitive driver gene.

## Introduction

As a drive gene mutation, anaplastic lymphoma kinase (ALK) gene rearrangement (ALKr) accounts for 2% to 7% of all cases of non-small-cell lung cancer (NSCLC) (1). Therefore, agents targeting ALKr might precisely treat this subtype of NSCLC. As a first-generation drug targeting ALKr, crizotinib has proven effective in patients with NSCLC harboring ALKr (2). However, most patients experience tumor recurrence within 1 year after crizotinib therapy. Moreover, brain metastasis (BM), which remains a substantial cause of morbidity and mortality, is the most common type of recurrence (3). The treatment of BM of ALK-positive NSCLC requires a multidisciplinary approach, including targeted therapy, chemotherapy, and radiotherapy. To date, no definitive treatment has been established. Haihong et al. reported that patients with BM of ALK-positive lung adenocarcinoma had better overall survival following tyrosine-kinase inhibitor (TKI) treatment or cranial radiotherapy. Moreover, cranial radiotherapy plays an important role in the treatment of these patients (4). A lack of response to radiotherapy has not been previously reported in patients with BM of ALK-positive lung adenocarcinoma. In this study, we found that alectinib was effective in a patient with BM of NSCLC refractory to radiotherapy that was negative for ALKr after resistance to crizotinib. In addition, we discuss the effects of different treatments for BM of ALK-positive lung adenocarcinoma by reviewing the relevant literature.

## Case Description

**Figure 1:**
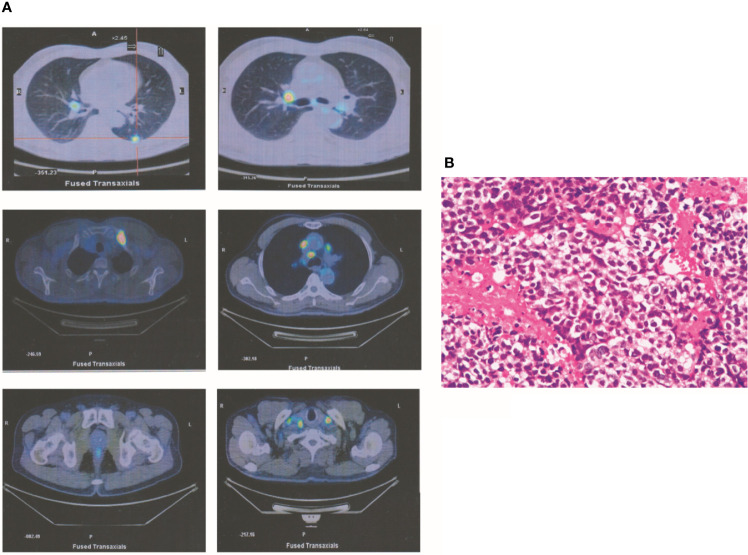
A T1cN3M1b NSCLC patient. **(A)** PET revealed that one occupying lesion is in left lung. There is one right supraclavicular lymph node enlargement and multiple mediastinal lymphadenopathies. Moreover, there were multiple bone metastases, including the fourth cervical vertebra, the left first rib, and the left pubis. **(B)** According to the immunohistochemical analysis, a diagnosis of poorly differentiated adenocarcinoma was made.

A 67-year-old man had a greater than 20-year history of smoking 20 cigarettes/day, although he quit smoking 10 years before presentation. In July 2015, he sought medical advice for right supraclavicular lymph node enlargement. Physical examination identified right supraclavicular lymph node enlargement that was not painful. Laboratory data were normal excluding elevation of carcinoembryonic antigen (CEA) levels (8.7). Positron emission tomography (PET) revealed one occupying lesion in the left lung, as well as right supraclavicular lymph node enlargement and multiple mediastinal lymphadenopathies. Moreover, there were multiple bone metastases, including metastases in the fourth cervical vertebra, left first rib, and left pubis (**Figure 1A**). According to the eighth edition of the classification of lung cancer, the stage of this malignancy was IV (T1cN3M1b). Transcutaneous needle biopsy was performed at the site of right supraclavicular lymph node enlargement. Based on the result of immunohistochemical analysis, a diagnosis of poorly differentiated adenocarcinoma was made (**Figure 1B**). Via next-generation sequencing (NGS) and fluorescence *in situ* hybridization, ALKr was detected in biopsy samples. Therefore, crizotinib therapy was started, which resulted in the shrinkage of all lesions after 3 months. In addition, CEA levels had declined to 6.7 at this time. At the end of the follow-up period, all lesions exhibited further shrinkage excluding the left lung lesion, which was stable. Laboratory analysis revealed normal CEA levels (<5.0).

**Figure 2:**
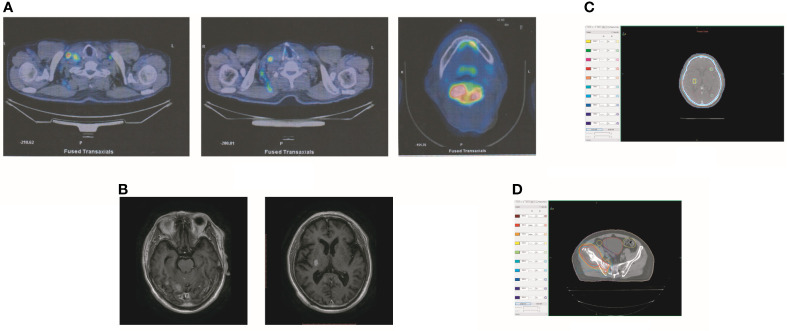
Tumor recurrence after treatment of crizotinib. **(A)** PET revealed multiple bone metastases in the right ilium and the right ischium. Moreover, there were multiple metastatic focuses in the brain. **(B)** The contrasted MR showed that the patient had BM. **(C)** The dose distribution of radiotherapy in BM. **(D)** The dose distribution of radiotherapy in the lesion of the right iliac bone.

**Figure 3:**
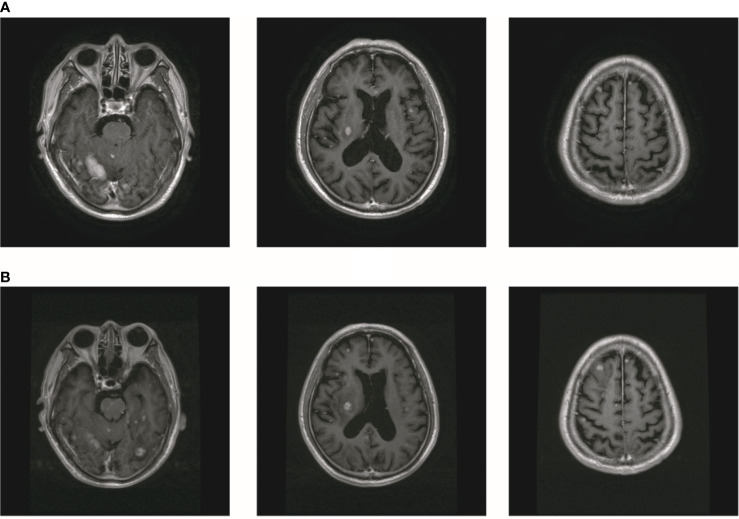
The number of BM increased after radiotherapy. **(A)** The MR showed that the number of brain metastasis increased to about 50. **(B)** MR showed that the number of BM increased to about 80.

**Figure 4:**
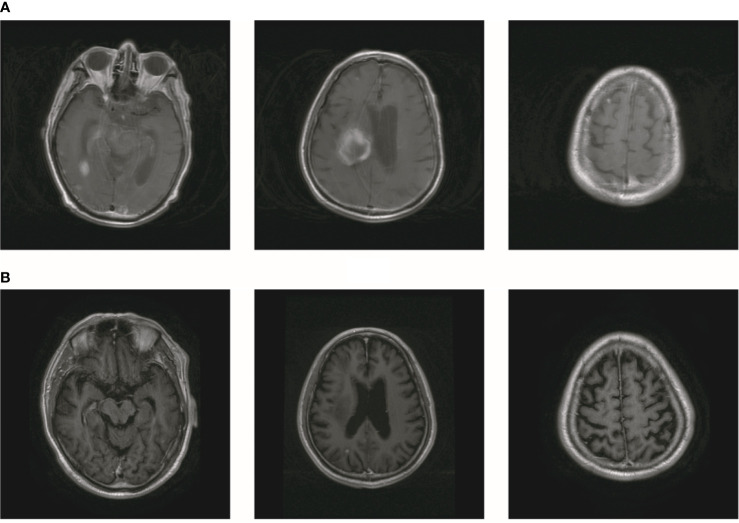
The number of BM decreased after treatment of alectinib. **(A)** MR showed that the number of brain metastasis decreased to about 60. **(B)** The number of brain metastasis further decreased to about 20.

In October 2019, the patient experienced dizziness and right hip pain. PET revealed multiple bone metastases in the right ilium and right ischium (**Figure 2A**). Moreover, there were multiple metastatic foci in the brain (**Figure 2A**). Contrast-enhanced magnetic resonance imaging (MRI) disclosed nine BMs (**Figure 2B**). Laboratory analysis revealed a CEA level of 3.6. We recommended biopsy of the right iliac bone. Because the patient had high intracranial pressure and worsening dizziness, he and his family refused biopsy. ALKr was not detected in a blood sample examined *via* NGS. Following Liu’s report (5), the patient received whole-brain radiotherapy (WBRT) combined with simultaneous integrated boost (SIB) directed at the metastatic foci. The dose of WBRT was 39.6 Gy delivered in 22 fractions, and that of SIB was 55 Gy delivered in 22 fractions (**Figure 2C**). The patient also received radiotherapy of the right iliac bone lesion (60 Gy in 24 courses, **Figure 2D**). Although radiotherapy resulted in improvement of the patient’s right hip pain, his intracranial pressure worsened. The patient underwent MRI after completing 10 fractions of cranial radiotherapy. MRI indicated that the number of BMs had increased to approximately 50 (**Figure 3A**). After careful consideration, we decided to finish WBRT. After finishing WBRT, the patient became comatose, and MRI revealed approximately 80 BMs, including some in the brain stem (**Figure 3B**). Satoh et al. reported that the overall concordance rate of the ALK status was 100% according to immunostaining between histologic and paired liquid-based cytology specimens (6). However, Aldea et al. found that the detection rate of genomic alterations was lower in patients with isolated central nervous system (CNS) progression (7). Because the result of blood testing was doubtful, the patient received alectinib 600 mg twice daily. After a week of oral alectinib treatment, the patient gradually regained consciousness, and his physical symptoms had gradually improved after a month of therapy. MRI revealed a reduction in the number of BMs to approximately 60 and a decrease in the total metastatic tumor volume (**Figure 4A**). Some metastatic foci vanished, especially in the brain stem. However, the right thalamus hemorrhaged and ruptured into the ventricle because the patient autonomously terminated antihypertensive therapy, resulting in cerebral hemorrhage. The patient was treated for cerebral hemorrhage. Meanwhile, the patient continued to take alectinib. After a month of treatment following the development of hemorrhage, the patient’s condition had gradually stabilized. MRI illustrated that most of the hematoma was absorbed and the number of BMs had further decreased to approximately 20 (**Figure 4B**). The total metastatic tumor volume had also further decreased.

## Discussion and Conclusions

BM, which is a frequent complication in patients with NSCLC, is associated with poor survival outcomes, and it poses clinical challenges for oncologists (8). At the initial diagnosis, 10% of patients with NSCLC have BM, and the brain is the only site of tumor relapse in 50% of patients with NSCLC (9). However, the risk of BM is higher in patients with NSCLC harboring ALKr. The rate of BM is approximately 20% in patients with NSCLC harboring ALKr at the initial diagnosis and up to 75% in patients after resistance to crizotinib (10). Therefore, the therapeutic effect on BM plays an important role in prolonging overall survival and improving patient quality of life. However, there is no standard treatment for BM of NSCLC. Prior studies used different strategies to treat BM of NSCLC, including surgery, radiotherapy, chemotherapy, targeted therapy, immunotherapy, and combinations of different modalities (11–15).

Radiotherapy, including WBRT (13, 16, 17) and stereotactic radiosurgery (SRS) (16–18), plays an important role in treating BM of NSCLC harboring driver gene mutations. In accordance with National Comprehensive Cancer Network recommendations, the patient received WBRT. To increase local control, we used SIB to increase the dose delivered to metastatic foci. However, during the course of radiotherapy, the number of BMs inexplicably increased in this patient. This phenomenon has not been reported previously. Prior studies mainly found that WBRT impairs cognitive function and quality of life and cited SRS as an alternative therapy for patients with BM. Some studies reported that SRS achieved good local control and resulted in less cognitive deterioration in patients with one to three BMs (19, 20). Hughes et al. reported that SRS alone could be adapted to treat patients with five to 15 BMs (21). Recently, Robin et al. found that patients with BM and ALKr could uniquely benefit from SRS (20). Thus, SRS alone may become a preferred strategy for treating BMs.

NGS did not identify ALKr in a blood sample from the patient. Therefore, the patient did not continue in using ALK inhibitor. Because of the poor accumulation of crizotinib in the CNS, many NSCLC patients with ALKr frequently develop BM after treatment with the drug. Second-generation ALK inhibitors, such as alectinib, can achieve a higher concentration in the CNS, resulting in enhanced efficacy against BM in NSCLC patients with ALKr (22). After crizotinib failure in patients with ALK-positive NSCLC, Novello et al. reported that alectinib had significantly better efficacy against BM than chemotherapy (3). It is unclear whether the combination of radiotherapy and TKIs has better efficacy against BM than either treatment alone. In a meta-analysis, Singh et al. reported no significant difference in efficacy between combined radiotherapy and TKI therapy and radiotherapy alone. Similarly, there was no significant difference in median overall survival between the TKI, radiotherapy, and combination alternatives (23). Thus, treatment should be selected according to the specific situation of the patient. In our case, the patient empirically received alectinib after radiotherapy failure, and unexpected success was achieved.

In summary, some new insights were revealed in this study. First, liquid biopsy is complementary to tissue biopsy in patients with NSCLC, mainly in those with EGFR mutation. However, ALK rearrangement should be assessed using tissue biopsy as much as possible. Second, brain metastasis of NSCLC might respond to second-generation TKIs, such as alectinib and ceritinib, after resistance to crizotinib regardless of the presence or absence of ALK rearrangement in liquid biopsy. Finally, combined radiotherapy and TKI therapy appears optimal in patients with brain metastasis of NSCLC after resistance to crizotinib in the absence of a definitive driver gene.

## Data Availability Statement

The original contributions presented in the study are included in the article/supplementary material. Further inquiries can be directed to the corresponding author.

## Ethics Statement

Written informed consent was obtained from the individual(s) for the publication of any potentially identifiable images or data included in this article.

## Author Contributions

CZ is responsible for all the work of this manuscript.

## Conflict of Interest

The author declares that the research was conducted in the absence of any commercial or financial relationships that could be construed as a potential conflict of interest.

## Abbreviations

NSCLC, non-small-cell lung cancer; ALK, anaplastic lymphoma kinase; ALKr, anaplastic lymphoma kinase gene rearrangement; BM, brain metastasis; TKIs, tyrosine-kinase inhibitors; CEA, carcinoembryonic antigen; NGS, next-generation sequencing; FISH, fluorescence *in situ* hybridization; WBRT, whole brain radiation therapy; SIB, simultaneous integrated boost; MR, magnetic resonance; CEA, carcinoembryonic antigen.
